# Supplementary material for: Mapler: a pipeline for assessing assembly quality in taxonomically rich metagenomes sequenced with HiFi reads
Source: Bioinformatics. 2025 Jun 6;41(6):btaf334. doi: 10.1093/bioinformatics/btaf334 (PMC12205171; doi:10.1093/bioinformatics/btaf334)
Supplement: btaf334_Supplementary_Data [file btaf334_supplementary_data.pdf]

Supplementary material for "Mapler: Assessing assembly quality in taxonomically-rich metagenomes sequenced with HiFi reads"

Nicolas Maurice[1,2], Claire Lemaitre[1], Riccardo Vicedomini[1] and Clémence Frioux[2]
[1]GenScale, Univ. Rennes, Inria, CNRS UMR 6074, Rennes, F-35000, France
[2]Inria, Univ. Bordeaux, INRAE, F-33400 Talence, France

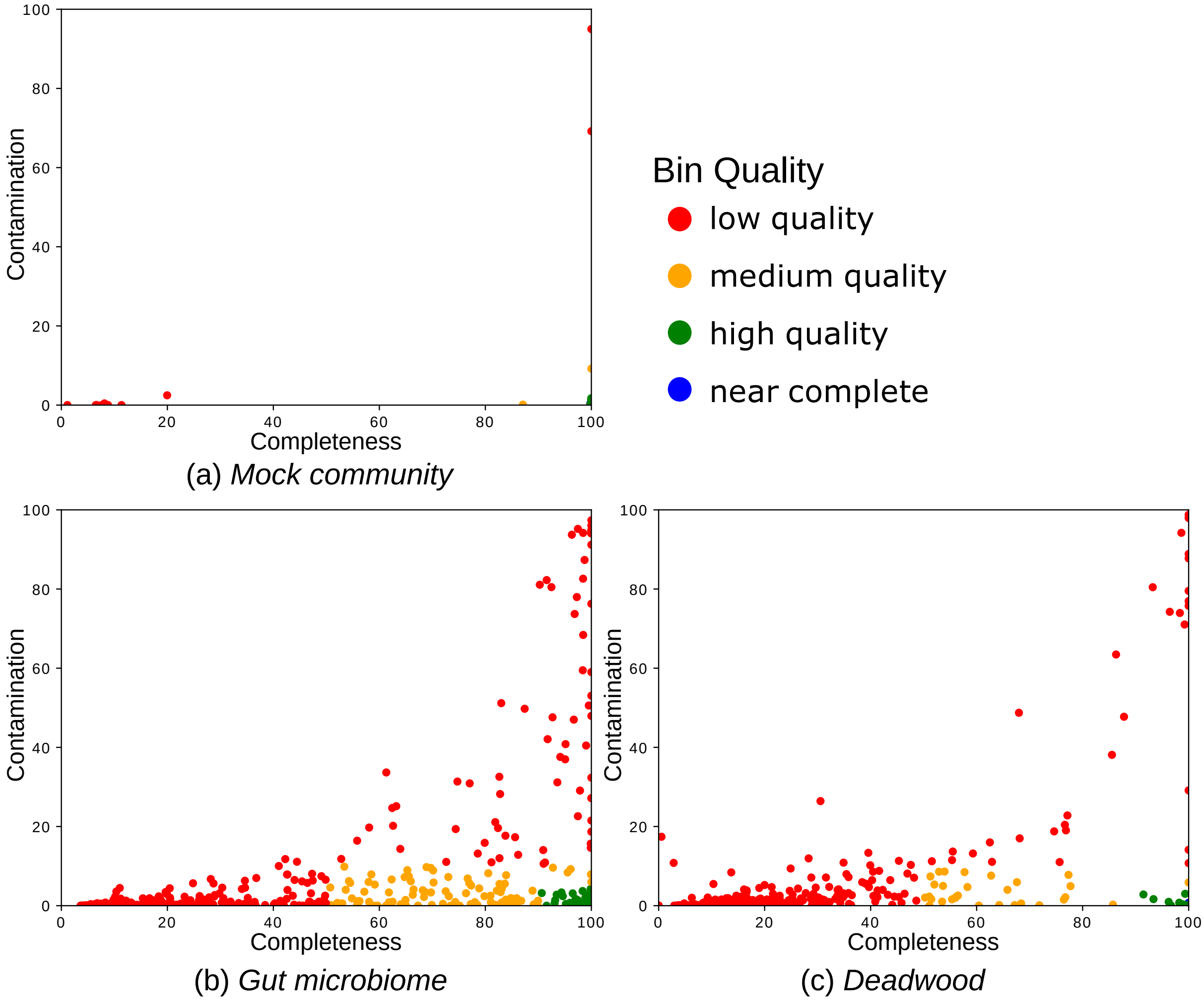

Figure S1: Comparison of the binning results between three samples, (a) mock community, (b) Gut microbiome, and (c) Deadwood, each assembled with metaMDBG. Green, orange and red dots denote the quality of the bin: high, medium and low respectively.

Rule	Threads	Memory (MB)	Time limit (hours)
metaquast	1	20,000	24
read_contig_mapping_evaluation	1	10,000	2
checkm	16	50,000	24
checkm_report_writer	1	50,000	1
kraken2_on_bins	12	100,000	4
gtdbtk_on_bins	20	50,000	8
read_contig_mapping_plot	1	30,000	2
extract_unmapped_reads	24	50,000	24
kraken2	12	100,000	4
kat_sect	12	200,000	4
fastqc	1	5,000	2

Table S1: Resource allocation for each snakemake evaluation rule used for the time evaluation of all analyses.

FastQC Report

Basic Statistics

Measure	Value
Filename	mapped_reads.fastq
File type	Conventional base calls
Encoding	Sanger / Illumina 1.9
Total Sequences	634609
Total Bases	11.7 Gbp
Sequences flagged as poor quality	0
Sequence length	1006-50047
%GC	59

Measure	Value
Filename	unmapped_reads.fastq
File type	Conventional base calls
Encoding	Sanger / Illumina 1.9
Total Sequences	231398
Total Bases	4.3 Gbp
Sequences flagged as poor quality	0
Sequence length	1000-48288
%GC	56

Figure S2: Statistics of assembled reads (”mapped reads”) and non-assembled reads (”unmapped reads”)from the metaMDBG assembly of the *Deadwood* sample, reported by FastQC.

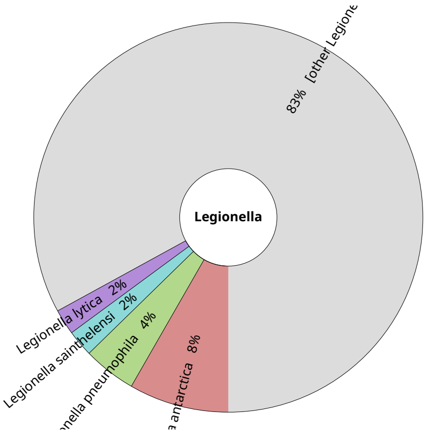

(a) Aligned *Legionella*-assigned reads

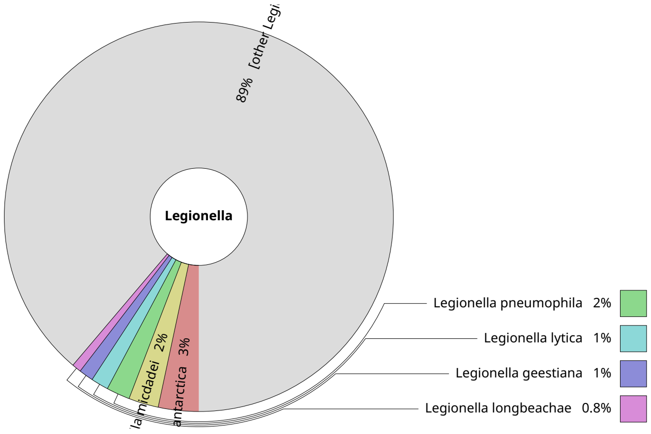

(b) Unaligned *Legionella*-assigned reads

Figure S3: Comparison of the taxonomic assessment of (a) aligned and (b) unaligned reads from the metaMDBG assembly of the *Deadwood* sample, with a focus on *Legionella* genus (0.05% of unaligned reads, 0.006% of aligned reads)

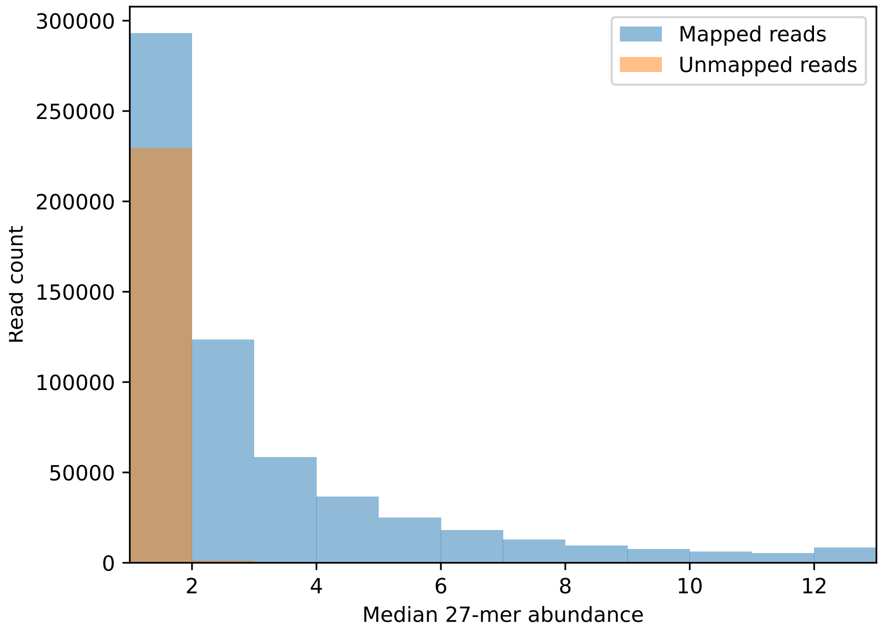

Figure S4: Comparative abundance of reads aligned (blue) or not aligned (orange, i.e. not assembled) to the metaMDBG assembly of the *Deadwood* sample

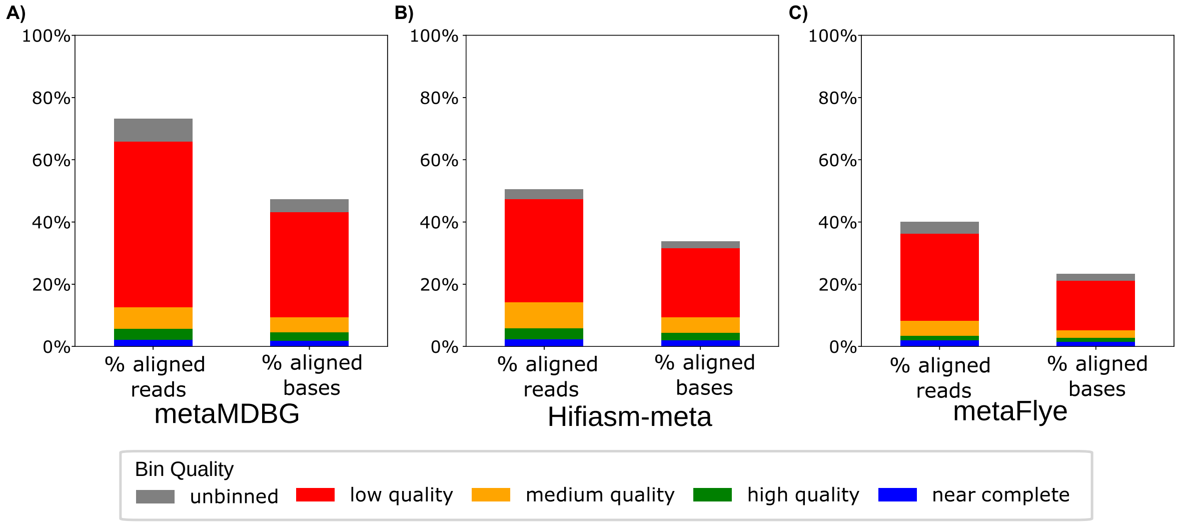

Figure S5: Comparison of the captured diversity for the *Deadwood* sample using three assemblers: (A) metaMDBG, (B) Hifiasm-meta, and (C) metaFlye.
